# Clinicopathological Significances of Cancer Stem Cell-Associated HHEX Expression in Breast Cancer

**DOI:** 10.3389/fcell.2020.605744

**Published:** 2020-12-23

**Authors:** Kexin Zhang, Qi Zhao, Zugui Li, Fangmei Fu, Hao Zhang, Junjie Fu, Minying Zheng, Shiwu Zhang

**Affiliations:** ^1^Nankai University School of Medicine, Nankai University, Tianjin, China; ^2^Department of Pathology, Tianjin Union Medical Center, Tianjin, China; ^3^Graduate School, Tianjin University of Traditional Chinese Medicine, Tianjin, China

**Keywords:** miRNA-HHEX-mRNA network, breast cancer, cancer stem cells, prognosis, HHEX

## Abstract

Aberrant expression of the transcription factor hematopoietic ally expressed homeobox/proline-rich homeodomain (HHEX/PRH) is implicated in numerous cancers. However, the association of HHEX with breast cancer (BC) remains unclear. In this study, HHEX mRNA and protein expression were analyzed using the Oncomine, UALCAN, GEPIA, TCGAportal, and HPA databases. We evaluated the effect of HHEX on clinicopathological parameters using Kaplan–Meier plotter, OncoLnc, TCGAportal, PROGgeneV2, and BC-GenExMiner. Western blotting was performed to compare the level of HHEX in breast samples of Tientsin Albino 2 mice, human breast precancerous lesions, benign breast tumors, and BC. The correlation between HHEX and cancer stem cells was investigated using the GEO (GSE52327 and GSE94865) and GEPIA datasets. Networks between HHEX and survival-related gene marker sets and microRNAs were analyzed using GEPIA, StarBase, and Cytoscape. Results of this study showed that HHEX expression in BC was significantly lower than those in breast precancerous lesions and benign breast tumors at both mRNA and protein levels. BC patients with lower HHEX expression had significantly worse overall survival and disease-free survival. Moreover, HHEX significantly affected the clinicopathology of BC. Specifically, low HHEX expression was correlated with the following groups of patients: age ≤51 years, ER-negative or PR-negative patients, HER-2 positive, triple-negative breast cancer, and basal-like BC. Immunohistochemical analysis of the breast samples showed significant differences of HHEX staining index (*P* < 0.001) among the three groups. To further investigate the mechanism, we determined the intersection of differentially expressed genes related to BC stem cells and those genes after HHEX expression was altered. This led to the identification of four potentially regulated genes-CXL12, BLNK, PAG1, and LPXN. Using StarBase and km-plotter, the negative regulation of HHEX expression and survival trends, including miR-130b, miR-30e, and miR-301b were joined into miRNA-HHEX-mRNA potential regulatory network. The abilities of proliferation, migration and invasion increased in MDA-MB-231 and BT-549 breast cancer cell lines after HHEX down expression and decreased after HHEX overexpression compared them in the control cells. In conclusion, these data suggest that HHEX expression is downregulated in BC and HHEX may regulate the development of BC through the stem cell-related genes.

## Introduction

Breast cancer (BC) is a high incidence cancer in women, and ~1.7 million new cases are reported globally each year (Siegel et al., 2020). BC development and progression occur through random alterations at the gene level, resulting in heterogeneous tumor cell populations. Cancer stem cells (CSCs) play an important role in tumorigenesis, metastasis, recurrence and drug resistance (Zhang et al., 2020b). BC stem cells (BCSCs) exist in epithelial-mesenchymal transition (EMT) and mesenchymal-epithelial transition (MET) states expressing different CSC markers. Moreover, EMT and MET states can be switched to each other, which endows BCSCs with high plasticity (Liu et al., 2014). In the EMT process, cancer cells detach from intercellular adhesion and become migratory and invasive (Shibue and Weinberg, 2017). It is necessary to recognize novel biomarkers related to EMT and CSCs in BC to improve prognoses and achieve the goal of individualized treatment of BC.

Haematopoietically expressed homeobox (HHEX) is also named as proline-rich homeodomain (PRH) is a homeodomain transcription factor involved in multiple developmental processes (Brickman et al., 2000; Martinez Barbera et al., 2000; Guo et al., 2003; Foley and Mercola, 2005), including cell proliferation and survival. HHEX dysregulation is associated with a variety of cancers (Soufi and Jayaraman, 2008; Gaston et al., 2016). BC cells and poorly differentiated hepatocellular carcinomas have low HHEX mRNA levels (Puppin et al., 2006; Su et al., 2012). HHEX inactivation occurs frequently in several cancer types, but the mechanisms of HHEX inactivation vary in different disease states. Researchers have shown that HHEX directly regulates the CD105 gene, which encodes the TGF β co-receptor protein endoglin (Kershaw et al., 2014), and goosecoid, a gene that induces EMT in a variety of tumor cells (Brickman et al., 2000; Williams et al., 2008). HHEX also regulates gene expression with multiple transcription factors, including c-Myc (Marfil et al., 2015) and SOX13 (Marfil et al., 2010). In addition, HHEX can interact with translation initiation factors, and modulate their activity and/or subcellular localization to regulate gene expression (Topcu et al., 1999; Topisirovic et al., 2003a,b; Marfil et al., 2015). In addition, TGF β signaling inactivates HHEX via multiple mechanisms, resulting in increased sensitivity to TGF β signaling (Marcolino et al., 2020). In summary, HHEX is related to EMT status and is a suppressor of tumorigenesis in BC. However, the correlations and mechanisms by which HHEX affects prognosis and CSCs in BCs remain unclear.

Here, we explored the multiple relationships between HHEX expression and BC. In the present study, we found that HHEX expression was lower in BC and correlated with prognosis and clinicopathology in BC patients. Tientsin Albino 2 (TA2) mice have a high incidence of spontaneous BC (SBC) (Du et al., 2020). SBC initiation associates with the infection of mouse mammary tumor virus and pregnancy (Du et al., 2020). HHEX expression was associated with an increase in the number of pregnancies, with a significant increase in the sixth pregnancy, followed by a decrease in SBC and reduced expression in normal mouse breast tissue. Immunohistochemical (IHC) analysis of human breast tumor tissues showed that precancerous lesions and benign breast tumors had higher staining index of HHEX compared with it in BC (*P* < 0.01) among precancerous lesions, primary breast carcinoma, and benign breast tumors. In addition, HHEX may regulate BC through the BC stem cell-related genes CXL12, BLNK, PAG1, and LPXN. In contrast to HHEX expression and survival trends, including miR-130b, miR-30e, and miR-301b, StarBase and km-plotter were joined into the miRNA-HHEX-mRNA potential regulatory network. Our findings suggest that HHEX is related to CSCs in BC, making it a promising biomarker for prognosis and individualized treatment of BC.

## Materials and Methods

### HHEX Expression Level Analysis

Oncomine (https://www.oncomine.org/), UALCAN (http://ualcan.path.uab.edu), GEPIA (http://gepia.cancer-pku.cn/detail.php), and TCGAportal (http://www.tcgaportal.org) were used to investigate the mRNA expression of HHEX in different tumor tissues and corresponding BC tissues. The Human Protein Atlas (https://www.proteinatlas.org/) database was used to examine HHEX protein expression in breast tissues. Next, we compared HHEX promoter methylation in BC patients with different molecular subtypes, using UALCAN (http://ualcan.path.uab.edu/). cBioPortal (www.cbioportal.org) is an open-access dataset for exploring multiple cancer genes. The Breast Invasive Carcinoma (TCGA, Nature 2012) dataset contains data from 463 cases, with pathologic diagnosis chosen by cBioPortal for further analyses of HHEX. The Wilcoxon rank sum test was used to assess the significance of the observed differences.

### Cancer Cell Lines and Cultures

The human breast cancer cell line BT-549 and MDA-MB-231 were obtained from the American Type Culture Collection (Manassas, VA, USA) and the culture conditions were described previously (Zhang et al., 2014).

### TA2 Mice With SBC

TA2 Mice were purchased from Tianjin Medical University. The female TA2 and male TA2 mice were raised for more than 16 months and euthanized according to the experimental requirements. These mice were divided into five groups according to the frequency of pregnancies, including the virgin TA2 mice (0); non-pregnancy (n), two pregnancies (2), four pregnancies (4), six pregnancies (6), and SBC. After sacrifice, the fresh BC tissues and mammary glands from the different groups were collected. The Institutional Animal Care and Use Committee of Tianjin Union Medical Center approved the animal experimentation protocols and all animal experiments were performed according to the Guidelines for the Care and Use of Laboratory Animals established by the Chinese Council on Animal Care.

### Transient Transfection

Three miRNA inhibitors and three mimics targeted miR-30e-5p, miR-301b, and miR-130b-3p of HHEX mRNA sequences were synthesized by Gene-pharma (Shanghai, China). The detailed information is provided in the Supplementary Tables 1, 2, and Supplementary Materials and Methods.

### Cell Migration and Invasion Assay

Transwell assays were used to evaluate the migration and invasion abilities of MDA-MB-231 and BT-549 cells transfected with and without miRNA inhibitors, mimics, and performed as described previously (Fei et al., 2019). The detailed information about these assays was provided in Supplementary Materials and Methods.

### Plate Clone Formation Experiment

Plate clone formation experiment was used to evaluate the proliferation ability of MDA-MB-231 and BT-549 cells transfected with and without miRNA inhibitors, mimics and performed as described previously (Liu et al., 2020). The detailed information is provided in Supplementary Materials and Methods.

### Western Blot Assay

The HHEX expression of MDA-MB-231 and BT-549 cells transfected with and without miRNA inhibitors, mimics, as well as tissue samples were determined by western blot. The detailed information is provided in Supplementary Materials and Methods. All western blot analyses were repeated three times independently.

### Breast Tissue Samples

Paraffin-embedded human breast (*n* = 145) tumor tissue samples were obtained from the Department of Pathology of Tianjin Union Medical Center. Breast tumor tissue samples from 145 cases were divided into the following three groups: 53 cases of precancerous lesions (group I), 44 cases of primary BC (group II), and 48 cases of benign breast tumors (group III). The Hospital Accreditation Committee of Tianjin Union Medical Center approved this study and the confidentiality of patient information was maintained.

### Immunohistochemical Staining, Scoring, and Quantification

IHC staining was carried out as described previously (Liu et al., 2020). The detailed information of staining, scoring, and quantification had been described in the Supplementary Materials and Methods.

### Relapse and Survival Analyses

Kaplan–Meier Plotter (http://kmplot.com/analysis/index.php?p=background) including 22,277 genes based on 1,809 patient microarrays was used to analyze correlations between HHEX expression and recurrence-free survival (RFS) and overall survival (OS). Kaplan–Meier survival plot, and hazard ratios and log rank *P*-values were used to analyze the two patient cohorts. Moreover, patients with BC were divided into two groups according to the median value of HHEX expression (high vs. low expression), the prognostic value of HHEX was calculated using OncoLnc (https://www.oncolnc.org/). TCGAPortal (http://www.tcgaportal.org) and The PROGgeneV2 Pan Cancer Prognostics Database (http://genomics.jefferson.edu/proggene/) were also used to analyze the role of HHEX in BC (Goswami and Nakshatri, 2014).

### Clinicopathological Parameters of BC Patients Analyzed Using BC Gene-Expression Miner v4.2 (BC-GenExMiner v4.4)

BC-GenExMiner v4.4 is a statistical mining tool that incorporates 264 independent datasets and three classical functions: expression, prognosis, and correlation (Jezequel et al., 2012, 2013). The parameters included age, nodal status, estrogen receptor (ER), progesterone receptor (PR), epidermal growth factor receptor-2 (HER-2), Scarff–Bloom–Richardson (SBR) grade, and Nottingham prognostic index (NPI), etc. The prognosis module can offer potential novel prognostic markers of BC. The correlation of HHEX with CXL12, BLNK, PAG1, and LPXN was assessed using the correlation module.

### Identification of mRNAs and miRNAs That Target HHEX

GEO (http://www.ncbi.nlm.nih.gov/geo) is a high-throughput genomics database that includes microarray, gene expression, and chip data. Two expression profiling datasets [GSE52327 (Liu et al., 2014) and GSE94865] were obtained from GEO [GPL570[HG-U133_Plus_2] Affymetrix Human Genome U133 Plus 2.0 Array, and GPL18943 NimbleGen Human Gene Expression 12x135K Array [100718_HG18_opt_expr]].

The GSE52327 dataset contained eight ALDH+ and ALDH- cells each. The GSE94865 dataset contained a 12-array study in which PRH/HHEX was either knocked down or overexpressed in MCF7 cells. StarBase v3.0 (http://starbase.sysu.edu.cn/index.php) is an open-source platform to identify the interactions among miRNA-ncRNA, miRNA-mRNA, ncRNA-RNA, RNA-RNA, RNA binding protein (RBP)-ncRNA, and RBP-mRNA from CLIP-seq, degradome-seq, and RNA-RNA interactome data. These three databases were used to identify miRNAs that are likely to bind to HHEX mRNA. In addition, starBase v3.0 was used to analyze the associations between miRNAs and HHEX mRNA.

### Functional Enrichment Analysis and the miRNA-HHEX-mRNA Network of HHEX

STRING (https://string-db.org/cgi/input.pl) is a database which can predict the direct and indirect interactions of protein-protein. These interactions come from computational predictions, knowledge transfer between organisms, and other databases (Szklarczyk et al., 2019). In this study, STRING was utilized to create an interaction network between HHEX and other important proteins. We investigated all four proteins and three miRNAs as a network of HHEX interaction systems. Cytoscape 3.7.2 (https://cytoscape.org/) constructed and analyzed a miRNA-HHEX-mRNA network of these candidates (Smoot et al., 2011).

### Statistical Analysis

SPSS 23.0 software (IBM Corp., USA) was used to analyze the data. We analyzed the HHEX protein expression between groups using Student's *t*-test and ANOVA. Kruskal–Wallis tests were used to compare the differences of HHEX expression level among the three BC groups. Mann–Whitney *U*-tests were used to analyze the expression levels of the four proteins between the BC groups. *P* < 0.05 was considered as statistical significance.

## Results

### HHEX Expression Levels in Pan-Cancer and BC Patients

The flowchart for this study is shown in Supplementary Figure 1. A summary of the databases used in this study is shown in Table 1. We first analyzed HHEX expression at the mRNA and protein level, and then analyzed its impact on clinicopathological parameters of BC. In further mechanistic exploration, we selected BC stem cell-related data sets to analyze the upstream and downstream mechanisms of HHEX.

**Table 1:** Summary of databases used in this study.

**Name**	**Link**	**This study**	**Keywords**
Oncomine	https://www.oncomine.org/resource/main.html	To analyze the HHEX mRNA expression in different cancers	Gene expression
UALCAN	http://ualcan.path.uab.edu/	To analyze the HHEX mRNA expression in different molecular subtypes of BC patients	Gene expression; 16 cancer types; survival curve; DNA methylation
GEPIA	http://gepia.cancer-pku.cn/detail.php	To assess the correlations between genes	Gene expression; survival curve; isoform details; genes correlation; similar genes detection
TCGAportal	http://www.tcgaportal.org	To investigate the expression and survival of HHEX in human tissues	Gene expression; 28 cancer types; survival curve; DNA methylation; mutation
Human Protein Atlas	https://www.proteinatlas.org/	To detect HHEX protein expression and survival	Proteins distribution; subcellular localization; impact for survival
Kaplan-Meier Plotter	http://kmplot.com/analysis/index.php?p=background	To analyzed the correlations between genes and miRNAs expression and RFS as well as OS	Breast cancer; subtype; survival curve
OncoLnc	https://www.oncolnc.org/	To detect HHEX survival	Breast cancer; survival curve
PROGgeneV2	http://genomics.jefferson.edu/proggene/		Gene expression; breast cancer; subtype; survival curve
bc-GenExMiner	http://bcgenex.centregauducheau.fr/BC-GEM/GEM-Accueil.php?js=1	To compute breast cancer gene-expression correlation analyses, for gene prognostic analyses	Gene-expression correlation; gene prognostic analyses
cBioportal	http://www.cbioportal.org/	To detect HHEX genetic alterations	Genetic alterations; survival curve
GEO	https://www.ncbi.nlm.nih.gov/geo/	To selected data sets related to breast cancer stem cells to explore further mechanisms	Gene expression; datasets details
STRING	https://string-db.org/cgi/input.pl	To obtain the interaction network between HHEX and other important proteins	Protein details; interactive network; functional enrichment
starBase v3.0	http://starbase.sysu.edu.cn/index.php	To predict the miRNAs related to HHEX; To perform miRNAs as well as correlation analysis between miRNAs and HHEX mRNA	miRNA target; Pan-cancer

**Figure 1:**
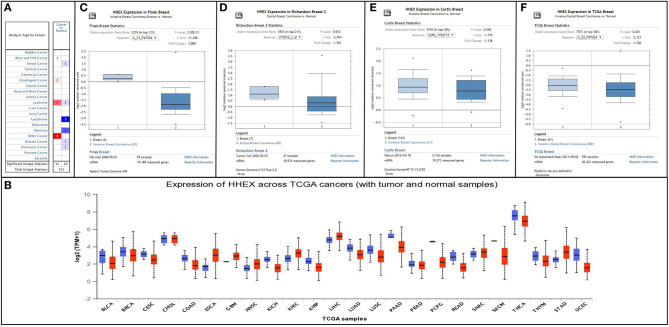
Expression levels of HHEX in pan-cancers and breast cancer (BC) patients. **(A,B)** Transcriptional levels of HHEX in different types of cancers determined using the Oncomine and UALCAN databases. Cell color was determined using the best gene rank percentile for the analyses within the cell, and the gene rank was analyzed using percentile of the target gene in the top of all genes measured in each study. **(C–F)** mRNA levels of HHEX in BC determined using Oncomine.

**Figure 2:**
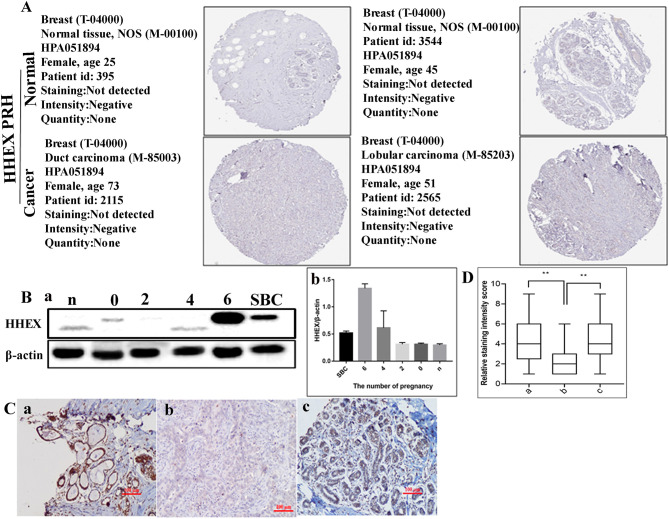
HHEX expression in human breast cancer (BC) tissues. **(A)** HHEX protein expression in BC tissues determined using HPA. **(B)** Expression levels of HHEX. (a) Expression levels of HHEX by western blot analysis in TA2 mice with different numbers of pregnancies and SBC. (b) Histogram shows the quantitative results of the HHEX expression in breast tissue TA2 mice with different numbers of pregnancies and SBC. **(C)** Immunohistochemical (IHC) staining of HHEX expression in human breast tumors (200×). (a–c) HHEX expression in group I–III, respectively. **(D)** Interquartile spacing diagram showing the quantitative results of the HHEX expression in the three groups. a: precancerous lesions; b: breast cancer; c: benign breast tumors. n, breast tissues of virgin TA2 mice; 0, breast tissues of TA2 mice without pregnancy; 2, breast tissues of TA2 mice with 2 pregnancies; 4, breast tissues of TA2 mice with 4 pregnancies; 6, breast tissues of TA2 mice with 6 pregnancies;. SBC, spontaneous breast cancer. Statistically significant differences are indicated: **P < 0.001.

Initially, we used Oncomine and UALCAN databases to compare the transcription levels of HHEX in many types of cancer and normal individuals. The HHEX mRNA expression was reduced in a variety of malignant tumors, including breast cancer (Figures 1A,B). Next, we assessed the transcript levels of HHEX in BC and normal tissues using Oncomine, GEPIA, and TCGAportal (Figures 1C–F, Supplementary Figures 2A,C). The transcript levels of HHEX in BC patients were significantly lower than those in normal tissues. Regarding HHEX protein expression in BC, we found that immunohistochemical analysis of HHEX was negative in BC and normal tissues (Figure 2A). These results showed that HHEX expression was lower in BC, thereby suggesting that it may be a tumor suppressor in BC.

### Expression of HHEX in TA2 Mice With Different Numbers of Pregnancies and SBC

To clarify the expression difference in the development of TA2 SBC, western blotting was used to detect HHEX protein expression in breast tissue from TA2 mice with different frequency of pregnancies and SBC (Figure 2Ba, Supplementary Figures 4A–C). The results confirmed that HHEX expression was the highest in TA2 breast tissue with 6 pregnancies compared with the level in the breast tissue of TA2 mice with non-pregnancy, 2 pregnancies, 4 pregnancies, and SBC. The difference of HHEX expression in breast tissue of TA2 mice with different pregnancies and SBC had statistical significance (*F* = 26.93*, P* < 0.001) (Figure 2Bb, Supplementary Figures 3, 4D). Breast tissue of TA2 mice with six pregnancies was proved to be precancerous lesion based on H&E staining (the result has been published in another paper (Du et al., 2020). Moreover, HHEX expression in SBC was downregulated (Supplementary Table 3).

### HHEX Expression in Human BC Tissues

To further quantify the role of HHEX in the development of BC, we collected 145 formalin-fixed and paraffin-embedded human breast samples including 53 cases of precancerous lesions (group I), 44 cases of primary BC (group II), and 48 cases of benign breast tumors (group III) and performed IHC staining. The differences of HHEX staining index among the three groups showed significant positive staining rates of HHEX (*P* < 0.001) and was the least in BC (Table 2A). After a paired comparison, HHEX expression was found to be significantly higher in group I than in group II (*P* < 0.001, Table 2B), and HHEX expression was higher in group III than in group II (*P* < 0.001, Figures 2C,D, Table 2C).

**Table 2A:** The differences of HHEX staining index among 53 cases of precancerous lesions (group I), 44 cases of primary BC (group II), and 48 cases of benign breast tumors (group III).

**Group**	***n***	**Staining index for HHEX**	**Value of statistic**	***P***
Group I	53	4.19 ± 2.067	χ^2^ = 25.081	0.001
Group II	44	2.11 ± 1.224		
Group III	48	4.77 ± 2.363		

**Table 2B:** The difference of HHEX staining index between among 53 cases of precancerous lesions (group I) and 44 cases of primary BC (group II).

**Group**	***N***	**Staining index for HHEX**	***Z***	***P***
Group I	53	4.19 ± 2.067	−4.139	0.001
Group II	44	2.11 ± 1.224		

**Table 2C:** The difference of HHEX staining index between 44 cases of primary BC (group II) and 48 cases of benign breast tumors (group III).

**Group**	***n***	**Staining index for HHEX**	***Z***	***P***
Group II	44	2.11 ± 1.224	−4.641	0.001
Group III	48	4.77 ± 2.363		

### Prognostic Value of HHEX in BC

**Figure 3:**
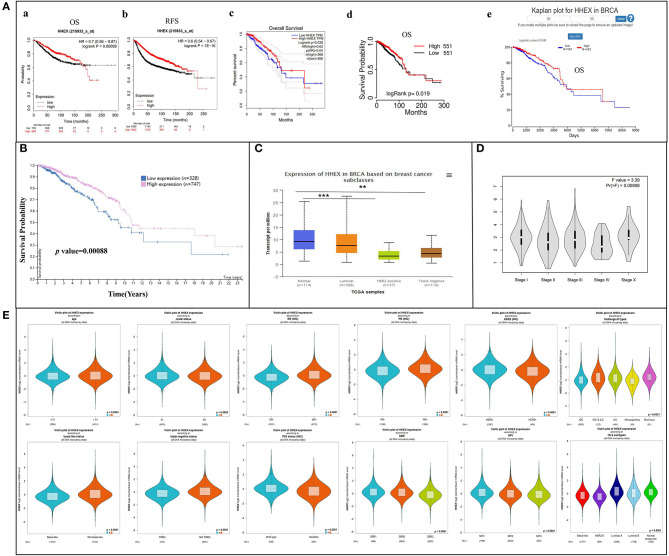
Prognostic value of HHEX in breast cancer (BC) and relationship between HHEX and clinicopathological parameters of BC patients. **(A)** Kaplan–Meier plotter, GEPIA, TCGAportal, and OncoLnc reveal the prognostic value of HHEX in BC. (a) Kaplan–Meier plotter reveals the overall survival curves based on the mRNA level of HHEX in BC patients. (b) Kaplan–Meier plotter reveals relapse-free survival curves based on the mRNA level of HHEX in BC patients. (c) Overall survival curves of HHEX in BC patients determined using GEPIA. (d) Overall survival curves of HHEX in BC patients determined using TCGAportal. (e) Overall survival curves of HHEX in BC patients determined using OncoLnc. **(B)** Survival curves based on protein level of HHEX in BC patients determined using HPA. **(C)** Relationship between mRNA levels of HHEX with subclasses of BC determined using UALCAN. **(D)** Correlation between HHEX and the pathological stage of BC patients determined using GEPIA. **(E)** Relationship between mRNA levels of HHEX and clinicopathological parameters of BC patients determined using bc-GenExMiner. **P < 0.05, ***P < 0.001.

To further explore the involvement of HHEX on BC patient prognoses, we performed survival analysis for HHEX using km-plotter, OncoLnc, TCGAportal, and PROGgeneV2. Figure 3A showed that 5-year (60 months/1,800 days) survival rates were higher in patients with HHEX high expression group compared with low expression group, and most BC patients with decreased HHEX mRNA expression had worse OS and RFS (Figure 3A, Supplementary Figure 2D). Low mRNA expression of HHEX [HR 0.7 [0.56–0.87], *P* = 0.00099] was associated with worse OS and RFS for BC patients (Figure 3Aa). However, the prognosis of BC patients with high HHEX mRNA expression was worse when the follow-up time was more than around 200 months. Regarding the HPA database, lower HHEX protein expression was significantly associated with shorter survival rate (Figure 3B).

### Relationship Between HHEX and Clinicopathological Parameters of BC Patients

To estimate the value of HHEX in the clinicopathology of BC, we performed sub-group analysis on different prognostic parameters based on BC-GenExMiner. HHEX mRNA expression was much lower in the subjects aged ≤51 years (Figure 3E). Moreover, ER-positive or PR-positive patients showed significantly higher expression of HHEX than ER-negative or PR-negative patients (all *P* < 0.0001). Further, HER-2 positive BC patients showed lower HHEX expression than HER-2 negative BC patients *(P* < 0.0001). Triple-negative BC (TNBC) is more aggressive and is characterized by poor prognosis with negative expression of estrogen receptors, progesterone receptors, and HER2 (Zhang et al., 2018). As expected, in this study, TNBC and basal-like BC patients displayed significantly decreased expression of HHEX than the non-TNBC and non-basal-like patients (all *P* < 0.0001).

The SBR can evaluate tubule formation, nuclear characteristics of pleomorphism, and mitotic index (Bansal et al., 2014). NPI can be used to stratify patients into additional prognostic groups based on tumor size, lymph node metastasis, and tumor grade. The SBR grade and NPI index are two useful prognostic models for BC (Lee and Ellis, 2008). According to the SBR criterion, we found a significant relationship between HHEX expression and the tumor grade. Reduced HHEX expression was observed in patients with poorly differentiated tumors (grades II and III) than in well-differentiated tumors. Patients with high NPI scores tended to express lower levels of HHEX (Figure 3E). Moreover, there is a significant correlation between the HHEX expression, histological types, and subtypes of BC. The subclass results of UALCAN were consistent with BC-GenExMiner (Figure 3C).

As for the pathological stage of BC patients, we used the GEPIA database to analyze HHEX. The results showed a significant correlation between HHEX expression and the pathological stage of BC (Figure 3D). Additionally, we also assessed the correlation between differentially expressed HHEX and clinicopathological profiles using the Kaplan–Meier database. As shown in Table 3, HHEX was significantly correlated with positive expression of ER, negative expression of HER2, and lymph node status. Both OS and RFS results indicated that HHEX was significantly associated with the HER2+ subtype. In summary, HHEX is a promising biomarker target for prognosis and is closely related to clinical factors of BC.

**Table 3:** Correlation between HHEX expression and clinicopathological features.

	**OS**		**RFS**	
**Characteristic**	**HR (95% CI)**	**Log rank *P***	**HR (95% CI)**	**Log rank *P***
**GENE SYMBOL: HHEX, affy id:215933_s_at**				
**ER status**				
ER positive	0.88(0.62–1.26)	0.4927	0.82(0.7–0.97)	**0.0191**
ER negative	0.78(0.49–1.23)	0.2826	0.82(0.65–1.02)	0.0792
**HER2 status**				
HER2 positive	1.52(0.74–3.12)	0.2518	0.83(0.54–1.28)	0.3982
HER2 negative	1.06(0.45–2.51)	0.8886	0.68(0.52–0.88)	**0.0038**
**Lymph node status**				
Lymph node positive	0.65(0.44–0.96)	**0.0278**	0.63(0.52–0.77)	**4.00E-06**
Lymph node negative	0.92(0.63–1.33)	0.6414	0.75(0.63–0.89)	**0.0009**
**Grade**				
1	1.25(0.52–3.02)	0.6169	0.86(0.51–1.45)	0.5758
2	0.78(0.5–1.2)	0.2502	0.73(0.57–0.93)	**0.0094**
3	0.85(0.61–1.18)	0.3365	0.9(0.72–1.12)	0.3396
**Intrinsic subtype**				
Basal	0.8(0.49–1.3)	0.3599	0.64(0.5–0.83)	**0.0006**
luminal A	0.72(0.5–1.02)	0.066	0.74(0.62–0.88)	**0.0006**
luminal B	0.94(0.65–1.36)	0.7459	0.69(0.57–0.84)	**2.00E-04**
HER2+	0.29(0.14–0.6)	**0.0004**	0.56(0.38–0.83)	**0.0033**

*The bold fonts means the values with statistical significance*.

### Promoter Methylation and Genetic Alterations of HHEX in BC Patients

**Figure 4:**
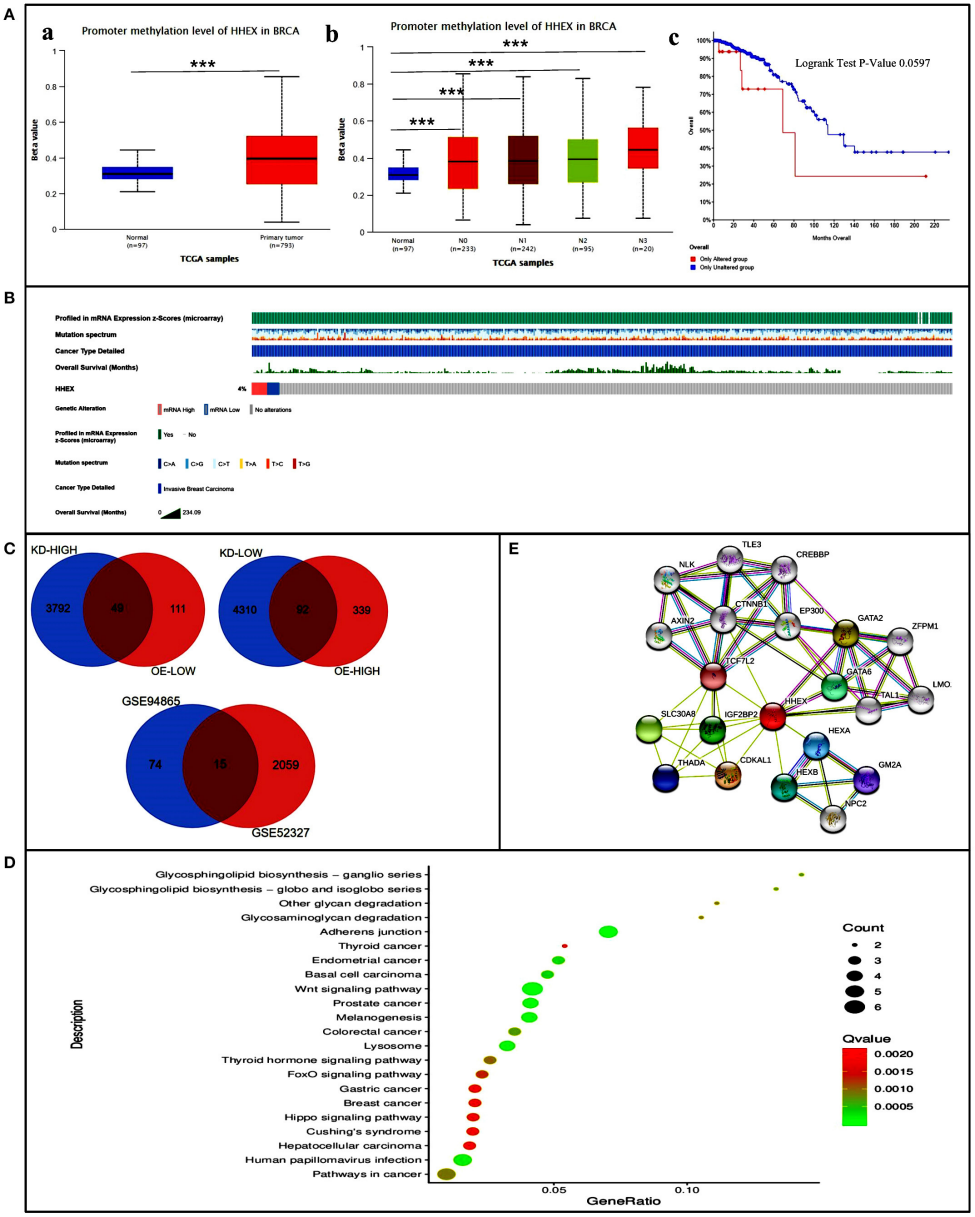
Promoter methylation and genetic alterations of HHEX and potential regulatory mechanism of HHEX in regulating breast cancer (BC). **(A)** Promoter methylation and genetic alterations of HHEX in BC patients. (a) Promoter methylation levels of HHEX in BC determined using UALCAN. (b) Relationship between nodal metastasis status of BC with the promoter methylation of HHEX determined using UALCAN. (c) OS for HHEX alterations was analyzed using cBioportal. **(B)** Genetic alterations of HHEX in BC patients determined using cBioportal. **(C)** Venn of the DEGs related to BC stem cells and the DEGs after HHEX changed. **(D)** KEGG pathway enrichment of the 15 common genes. **(E)** The neighbor gene network of 15 genes was constructed using STRING. ***P < 0.001.

To explore how HHEX affects the prognosis of BC, we first explored the methylation levels and genetic mutations of HHEX. Using UALCAN, we found that promoter methylation of HHEX was higher in BC than in the normal group (Figure 4Aa). Moreover, the nodal metastasis status of BC was significantly associated with promoter methylation of HHEX (Figure 4Ab). Meanwhile, as shown in Figure 4B, HHEX was altered in 4% of the queried BC patients. After analysis by Kaplan–Meier plot and log rank test, patients with HHEX alterations demonstrated shorter OS (Figure 4Ac).

### Potential Regulatory Mechanism of HHEX in Regulating BC

#### Identification of Differentially Expressed Genes (DEGs) Between EMT and MET States (GSE52327)

BCSCs can mediate metastasis, resist to radiation and chemotherapy, contribute to relapse and affect patient prognosis. Therefore, we selected data sets related to BCSCs to explore further mechanisms. A total of 2,074 DEGs were identified from GSE52327 in EMT compared with MET. Moreover, HHEX were one of the DEGs among the data sets (Supplementary Materials), indicating that it may be related to BCSCs.

#### Identification of DEGs Between Knockdown or Overexpression of HHEX (GSE94865)

To further explore the possible regulatory mechanism of downstream genes following altered HHEX expression, we selected the knockdown/overexpression data set for analysis. In order to ensure the consistency of the gene expression trend, we selected upregulated genes by knocking down HHEX and down regulated genes with overexpressed HHEX to obtain the common genes. Similarly, we selected downregulated genes by knocking down HHEX and upregulated genes with overexpressed HHEX. Subsequently, we obtained 49 and 92 genes that expressed the same trend changed between HHEX knockdown or overexpression, respectively (Figure 4Aa).

#### PPI Network and Kyoto Encyclopedia of Genes and Genomes (KEGG) Pathway Enrichment Analysis of Common DEGs

Next, to further investigate the mechanisms behind HHEX function, we took the intersection of the DEGs related to BCSCs and the DEGs following altered HHEX expression. From this, we obtained 15 common genes (Figure 4C). Subsequently, PPI network and KEGG pathway enrichment analyses were performed using STRING. Both network and KEGG pathways showed that HHEX is related to Wnt-related genes, including CTNNB1, AXIN2, and TCF7L2, and the Wnt signaling pathway itself (Figures 4D,E). The Wnt signaling pathway is a classic pathway for CSC regulation. These results indicate that HHEX may regulate BC via the Wnt signaling pathway.

#### Intersection of Four Survival-Related Genes and Their Correlations With HHEX

**Figure 5:**
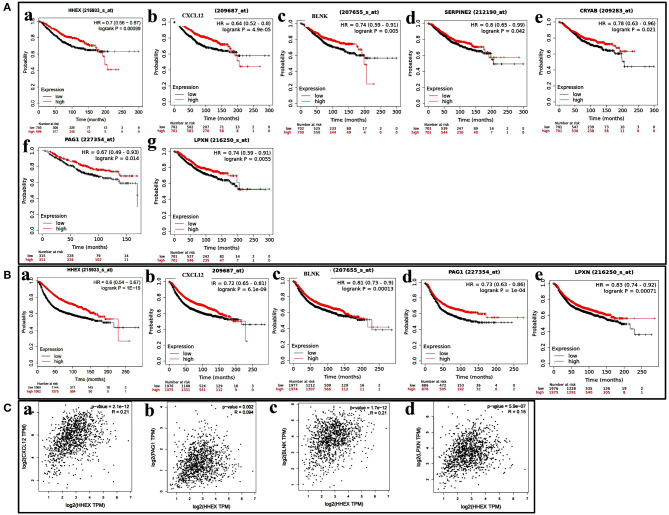
Potential regulatory mechanism of HHEX in breast cancer (BC). **(A,B)** Kaplan–Meier plotter reveals the overall survival and Relapse-free survival curves based on the mRNA level of CXL12, BLNK, PAG1, and LPXN in BC patients. **(C)** The positive correlation between HHEX expression and CXL12, BLNK, PAG1, and LPXN by GEPIA.

We further investigated the OS and RFS for the 15 genes. As shown in Figures 5A,B, four genes—CXL12, BLNK, PAG1, and LPXN—were significantly associated with survival. We further used the GEPIA database to analyze the correlation between HHEX and the four genes, and found them to have significant positive correlations (Figure 5C).

#### The Regulatory Networks of HHEX in BC

**Figure 6:**
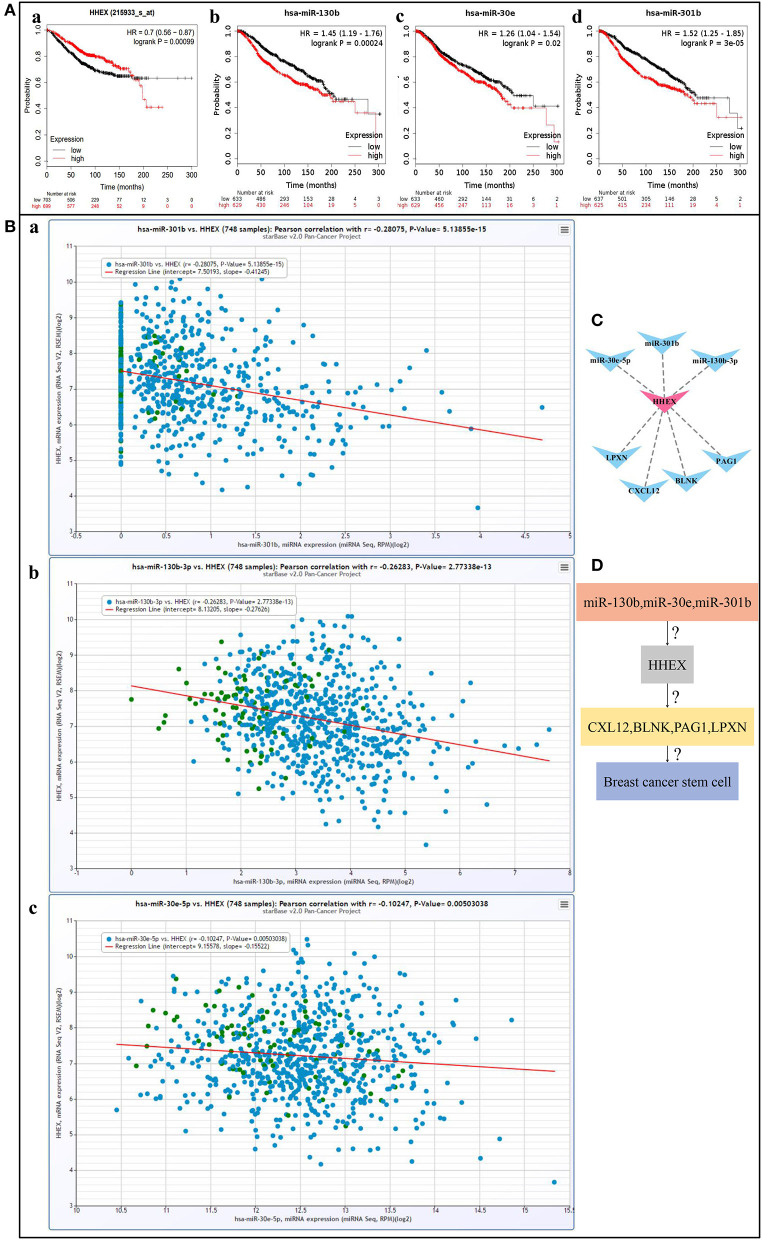
miRNA-HHEX-mRNA potential regulatory mechanism of HHEX in breast cancer (BC). **(A)** Overall survival curves of HHEX, miR-130b, miR-30e, and miR-301b in BC patients determined using Kaplan–Meier plotter. **(B)** The negative correlation between HHEX expression and miR-130b, miR-30e, and miR-301b by StarBase. **(C)** The miRNA-HHEX-mRNA potential regulatory network was constructed using Cytoscape. **(D)** The potential regulatory mechanism of HHEX in BC.

To further enrich the regulatory mechanism, we tried to find possible upstream regulatory mechanisms of HHEX by predicting miRNAs. Using StarBase and km-plotter, we found three miRNAs, including miR-130b, miR-30e, and miR-301b, which oppose HHEX expression and survival trends (Figure 6A). Moreover, Figure 6B shows that HHEX and three miRNAs had significant negative correlations. Next, we constructed the miRNA-HHEX-mRNA potential regulatory visualized in Figures 6C,D. This network may be one possible mechanism by which HHEX regulates BCSCs to affect BC prognosis.

### miRNAs Negatively Regulate HHEX Expression

**Figure 7:**
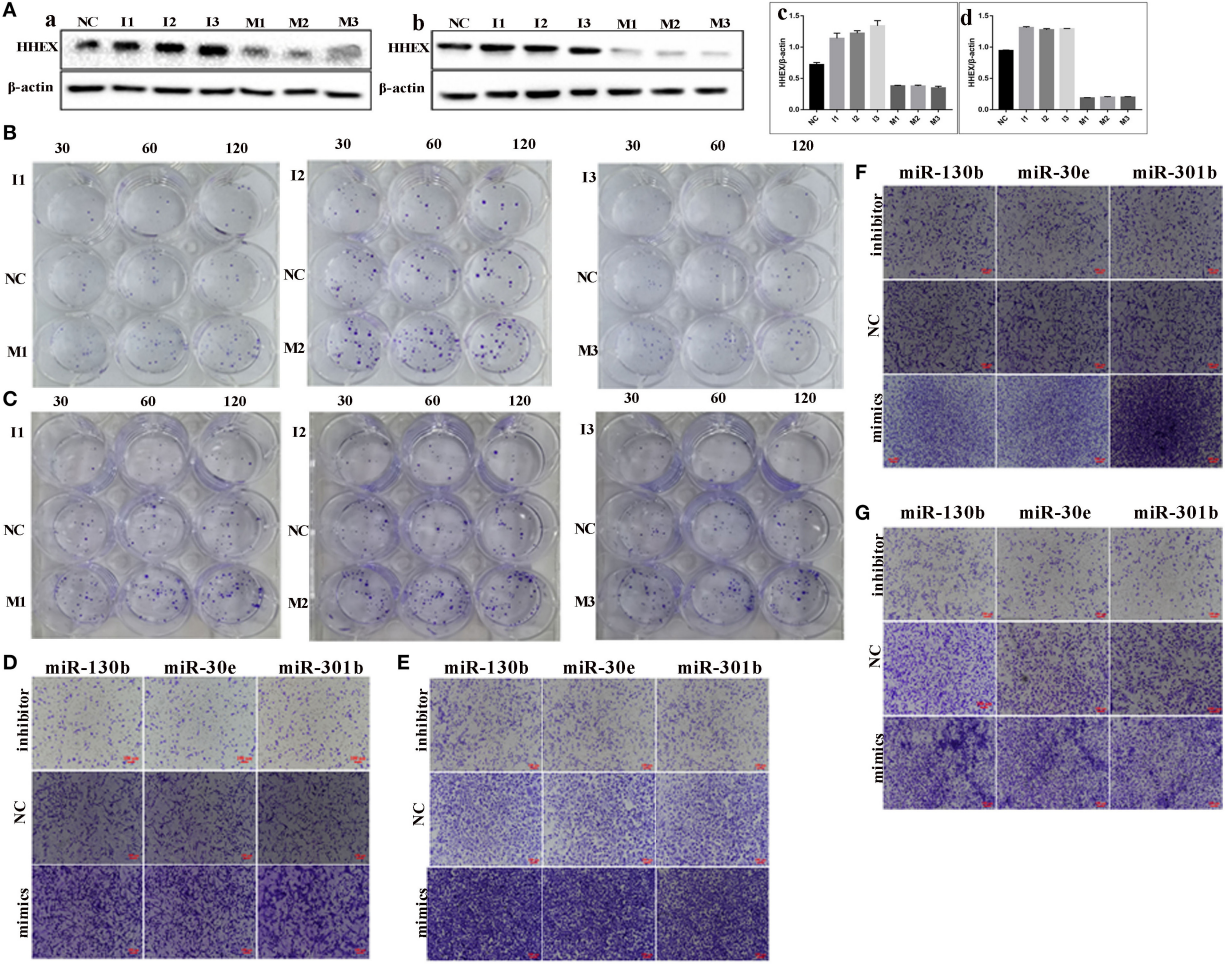
miRNAs negatively regulated HHEX and influenced the migration, invasion, and proliferation ability in MDA-MB-231 and BT-549. **(A)** HHEX expression of MDA-MB-231 and BT-549 transfected with and without miRNA inhibitors, mimics. (a) HHEX expression in MDA-MB-231. (b) HHEX expression in BT-549. (c) Histogram showed the quantitative result of the HHEX expression in MDA-MB-231 with different treatments. (d) Histogram showed the quantitative result of the HHEX expression in BT-549 with different treatments. **(B)** Plate clone formation experiment was used to compare the proliferation ability of MDA-MB-231 transfected with and without miRNA inhibitors, mimics. **(C)** Plate clone formation experiment was used to compare the proliferation ability of BT-549 transfected with and without miRNA inhibitors, mimics. **(D)** Transwell migration assay showed the cells migration capacity of MDA-MB-231 transfected with and without miRNA inhibitors, mimics (100×). **(E)** Transwell migration assay showed the cells migration capacity of BT-549 transfected with and without miRNA inhibitors, mimics (100×). **(F)** Transwell invasion assay of MDA-MB-231 cells transfected with and without miRNA inhibitors, mimics (100×). **(G)** Transwell invasion assay of BT-549 cells transfected with and without miRNA inhibitors, mimics (100×). NC, normal control; M1, miR-130b mimics; M2, miR-30e mimics; M3, miR-301b mimics; I1, miR-130b inhibitor; I2, miR-30e inhibitors; I3, miR-301b inhibitor.

The expression of HHEX was detected in MDA-MB-231 and BT-549 cells transfected three miRNA inhibitors and three mimics targeted miR-30e-5p, miR-301b, and miR-130b-3p of HHEX mRNA sequences. HHEX expressions of MDA-MB-231 and BT-549 after miR-30e-5p, miR-301b, and miR-130b-3p mimics treatment decreased compared with normal control (NC) and the differences had statistical significance (*F* = 221.5*, P* < 0.001). HHEX expressions of MDA-MB-231 and BT-549 after miR-30e-5p, miR-301b, and miR-130b-3p mimics treatment increased compared with NC and the differences had statistical significance (*F* = 6423.0*, P* < 0.001) (Figure 7A, Supplementary Figures 4E,F, Supplementary Tables 4, 5).

### The Migration, Invasion, Proliferation Abilities Decreased in Cells With HHEX Overexpression

miR-130b, miR-30e, miR-301b negatively regulated HHEX expression and the expression levels of the three miRNAs associated with the migration, invasion and proliferation abilities in MDA-MB-231 and BT-549. The migration ability of cells transfected with miRNA mimics was higher than that in NC (Figure 7). Results of plate cloning assay showed that the numbers of clones formed in MDA-MB-231 and BT-549 transfected with miRNA inhibitors were less than NC. The numbers of clones formed in MDA-MB-231 and BT-549 cells transfected with mimics were higher than NC (Figures 7B,C). Transwell migration assay showed that the number of migrated cells in cells transfected with miRNA inhibitors was lesser than that of NC. The number of migrated cells in miRNA mimics groups was more than that of NC (Figures 7D,E). Figures 7F,G reveals that the invasion ability in cells transfected with miRNA mimics was higher than that in NC. The invasion ability in cells transfected with miRNA inhibitors was lower than that in NC. The overexpression of HHEX could decrease the abilities of migration, invasion, and proliferation in MDA-MB-231 and BT549 cells, and the differences had statistically significant (Supplementary Tables 6–8).

## Discussion

BC is a common malignant tumor in women with relatively high rates of morbidity, and mortality as well as low rates of 5-year survival (Siegel et al., 2020). Due to our lack of understanding of BCSCs, the prevalence of heterogeneity in BC tissues resulted in limited value of many common prognostic predictors for BC recurrence, especially poor BC prognosis. HHEX is a tumor suppressor and can inhibit tumor metastasis (Soufi and Jayaraman, 2008; Gaston et al., 2016). The expression of HHEX level was associated the prognosis of patients with BC (Kershaw et al., 2017). In addition, TGF β signaling downregulates HHEX activity and induces an EMT that facilitates cell migration (Marcolino et al., 2020). HHEX induces promyelocyte self-renewal and cooperates with growth factor independence to cause myeloid leukemia in mice (Jackson et al., 2018). HHEX regulates the self-renewal of hematopoietic stem cell (HSC), emergency hematopoiesis, and the initiation of acute myeloid leukemia (AML) via repression of the Cdkn2a tumor suppressor locus (Jackson et al., 2017, 2020). Thus, HHEX may be a putative biomarker related to CSC and prognosis of BC. However, the links between HHEX, prognosis, and CSCs in BCs remains unclear.

Researchers have found that HHEX suppresses breast tumor growth (Kershaw et al., 2017). In both ductal and lobular breast carcinomas, a significant reduction in nuclear HHEX was observed (Puppin et al., 2006). Overexpression of HHEX inhibits cell migration and invasion by MDA-MB-231 breast cancer cells (Kershaw et al., 2014). In this study, we found that the mRNA and protein levels of HHEX in BC patients were significantly lower than those in normal tissues, which is consistent with previous data. Next, we used IHC to further verify HHEX expression. IHC analysis showed significant positive staining rates of HHEX (*P* < 0.001) among precancerous lesions, primary BC, and benign breast tumors. Moreover, low HHEX mRNA expression is associated with poor prognosis in BC (Kershaw et al., 2017). Adenovirus-mediated HHEX was used to transfect the hepatoma cell line (Hepa1–6) which could decrease the expression levels of c-Jun and Bcl 2, and increase some tumor suppressor genes (P53 and Rb) expression (Su et al., 2012). In our study, HHEX down-regulation correlated with a superior prognosis with respect to both OS and RFS in BC. In addition, the prognostic role of HHEX was significant regardless of age, stage, grade, lymph node, metastasis, ER status, HER2 status, lymph node status, and intrinsic subtype. Our findings that HHEX mRNA and protein are expressed at low levels in BC, strongly correlates with disease progression and confers significant survival advantage to patients with BC.

As for HHEX gene alternative and DNA methylation, results of Saied et al. showed that DNA methylation distribution and HHEX gene methylation could be used as a potential diagnostic marker for trisomy 8 AML (Saied et al., 2015). Few studies have examined the effect of HHEX mutations in BC. In our study, data analysis of UALCAN showed that promoter methylation of HHEX was higher in BC than in the normal group. Moreover, nodal metastasis status of BC was significantly associated with promoter methylation of HHEX. HHEX was altered in 4% of the queried BC patients with HHEX alterations that demonstrated shorter OS. While transcriptome sequencing can detect only static mutations, it cannot directly provide information on protein activity or expression. Therefore, the function of promoter methylation and genetic alterations of HHEX needs to be further studied in BC patients.

Recent findings have shown that CSCs are characterized by enhanced invasiveness and clonogenicity, and they play critical roles in metastatic dissemination and clinical relapse in a variety of cancers. Research has demonstrated that in prostate cells, HHEX regulates multiple genes involved in EMT and TGF β signaling (Marcolino et al., 2020). The dramatic enlargement of the endocardial cushions in the absence of HHEX is due to decreased apoptosis and dysregulated EMT (Hallaq et al., 2004). Thus, we investigated probable links between HHEX-regulated genes and CSC-like DEGs.

In GSE52327, BC cells from patients were sorted using flow cytometry to select cells that were ALDH+, whose profiles were compared with those of ALDH- cells (Liu et al., 2014). BCSCs exist in distinct EMT and MET states. Different EMT and MET state associated with the proliferative capacity and invasive characteristics of BCSCs. The plasticity of BCSCs allows them to switch between EMT and MET (Liu et al., 2014). Thus, we selected data sets to explore further association between HHEX expression and BCSCs. A total of 2,074 DEGs were identified from GSE52327 in status with EMT compared with MET. HHEX was one of the DEGs among the data sets, indicating that it may be related to BCSCs. Subsequently, we obtained 49 and 92 genes that showed similar expression trends for knockdown or overexpression of HHEX in GSE94865. Next, to further investigate the mechanism of HHEX, we analyzed the intersection of the DEGs related to BCSCs and the DEGs following abnormality in HHEX expression. Finally, we obtained the following 15 common genes: CCL5, CXCL12, BLNK, TFPI, CRISPLD2, DLC1, SERPINE2, TENM4, CRYAB, SRPX, PLAC8, KLHDC8B, MSX1, PAG1, and LPXN.

Among these genes, more than half have already been shown to be closely related to stem-like properties of tumor cells (Li et al., 2017, 2019; Taghiyar et al., 2017; Langer et al., 2018), and the mechanism involved in CSCs can be regulated through EMT, Wnt, and TGF-β1 signaling. CD133^(+)^ ovarian cancer stem-like cells promote the metastasis of CD133^(−)^ cancer cell metastasis via CCL5-induced EMT (Zeng et al., 2015). DLC-1 tumor suppressor inhibited TGF-β1 signaling and regulated CD105 expression in human non-small cell lung carcinoma cells (Zhang et al., 2020a). Further, inhibitor of DNA binding 1 (Id1) mediates the stemness of colorectal cancer cells through the Id1-c-Myc-PLAC8 axis via the Wnt/β-catenin and Shh signaling pathways (Sun et al., 2019) and upregulation of PAG1/CBP contributes to adipose-derived mesenchymal stem cell-promoted tumor progression and chemoresistance in BC (Lu et al., 2017). These genes have the potential to become CSC-related targets and should be addressed in follow-up studies using molecular biology techniques.

Interestingly, using bioinformatics-based algorithms for functional characterization, we also showed that HHEX is a targetable gene that has a high degree of gene neighborhood, and it is a probable modulator of survival genes, including BLNK, PAG1, and LPXN as well as known markers of cancer stemness, namely CXCL12, in patients with BC. In addition, our study identified several miRNAs that were associated with HHEX. These short (20–24 nt) non-coding RNAs, normally regulated the gene expression via post-transcriptional modification, have also been implicated in human carcinogenesis (Hayes et al., 2014). In fact, miR-30e-5p, miR-301b, and miR-130b-3p can be used as diagnostic and prognostic markers of BC. Specific miRNAs have been linked to BC development, proliferation, apoptosis resistance, invasion, and metastasis. SOX2 regulates the development of BC through the SOX2/miR-181a-5p and miR-30e-5p/TUSC3 axes (Liu et al., 2017). MicroRNA-301b promotes cell proliferation in TNBC by targeting CYLD (Song et al., 2018). MiR-130b-3p inhibits cell invasion and migration by targeting the Notch ligand Delta-like 1 in breast carcinoma (Shui et al., 2017). These findings indicate that HHEX may regulate BC prognosis by mediating the expression of other genes (CXCL12, BLNK, PAG1, and LPXN) and miRNAs (miR-30e-5p, miR-301b, and miR-130b-3p). In order to verify the relationships between miR-30e-5p, miR-301b, miR-130b-3p and HHEX expression, three miRNA inhibitors and three mimics targeted miR-30e-5p, miR-301b, and miR-130b-3p of HHEX mRNA sequences were used to transfect MDA-MB-231 and BT-549. When miR-30e-5p, miR-301b, miR-130b-3p was up-regulated, the expression level of HHEX was decreased and the abilities of cell migration, invasion and proliferation increased. While miR-30e-5p, miR-301b, miR-130b-3p was down-regulated, the expression level of HHEX was increased and the abilities of cell migration, invasion and proliferation decreased. Furthermore, we studied the mechanism involved in miR-30e-5p, miR-301b, miR-130b-3p and HHEX to BC development, proliferation, invasion, and metastasis. To sum up, miR-30e-5p, miR-301b, miR-130b-3p may regulate the development of breast cancer cells by regulating HHEX. However, the elucidation of a specific and precise mechanism requires further investigations. And we would like to verify their mutual relationship through experiments in the future work.

In summary, we have identified HHEX in BC through integrated bioinformatics analysis, indicating that it is a good target for BC prognosis and related to CSCs. Additionally, we experimentally verified that HHEX expression is downregulated in BC tissues compared with that in the breast tissue of TA2 mice with 6 pregnancies (precancerous lesion). Results of bioinformatics analysis from multiple databases suggest that the expression level of HHEX is significantly associated with OS and RFS, and HHEX may be a candidate biomarker and therapeutic target for BC. Further detailed study about the potential mechanism of HHEX regulating the development of BC is needed.

## Data Availability Statement

The original contributions presented in the study are included in the article/Supplementary Materials, further inquiries can be directed to the corresponding author.

## Ethics Statement

The studies involving human participants were reviewed and approved by Tianjin Union Medical Center. Written informed consent for participation was not required for this study in accordance with the national legislation and the institutional requirements. The animal study was reviewed and approved by Tianjin Union Medical Center. Written informed consent was obtained from the owners for the participation of their animals in this study.

## Author Contributions

SZ designed the study and interpreted data, contributed to manuscript writing, and approved the manuscript before submission. KZ and QZ collected and analyzed data and approved the manuscript before submission. ZL, FF, and HZ collected, analyzed, and interpreted the data and approved the manuscript before submission. MZ and JF collected data, gave constructive comments on the manuscript, and approved the manuscript before submission.

## Conflict of Interest

The authors declare that the research was conducted in the absence of any commercial or financial relationships that could be construed as a potential conflict of interest.

## Abbreviations

HHEX: haematopoietically expressed homeobox/proline-rich homeodomain
TA2: Tientsin Albino 2 mouse
SBC: spontaneous breast cancer
BC: Breast Carcinoma
DEGs: Differentially expressed genes
RBP: RNA binding protein
OS: Overall Survival
RFS: Relapse-Free Survival
ER: Estrogen Receptor
PR: Progesterone Receptor
HER2: Epidermal Growth Factor Receptor 2
SBR: Scarff-Bloom & Richardson
NPI: Nottingham prognostic index
CSC: Cancer stem cell
IHC: Immunohistochemical staining.
