# Supplementary material for: Clinicopathological Significances of Cancer Stem Cell-Associated HHEX Expression in Breast Cancer
Source: Front Cell Dev Biol. 2020 Dec 23;8:605744. doi: 10.3389/fcell.2020.605744 (PMC7785851; doi:10.3389/fcell.2020.605744)
Supplement: Supplementary file 1 [file Data_Sheet_1.doc]

Supplementary materials and methods

Transient miRNA transfection

Three miRNA inhibitors, three mimics targeted miR-30e-5p, miR-301b, and miR-130b-3p of HHEX mRNA sequences were synthesized by Gene-pharma (Shanghai, China). When the cancer cells were 60–70% confluence in six-well plates (2×10^5^ cells/well), Lipofectamine 2000 (Invitrogen, Carlsbad, CA, USA) and 1× Opti-MEM (Gibco, USA) were used to dilute the inhibitors and mimics siRNA following the manufacturer’s protocol, and the mixture was added to the cells. The cells before and after transfection were used to examine the expression of HHEX.

Cell migration and invasion assay

Transwell assays were used to evaluate the migration and invasion abilities of MDA-MB-231 and BT-549 cells transfected with and without miRNA inhibitors, mimics. For transwell migration assays, the cell culture inserts (8 μm, BD-Falcon, Franklin Lakes, NJ, USA) were placed into 24-well plates. The cells (5×10^4^ cells per insert) in 200 μL medium without FBS were added to the upper chambers, and the medium supplemented with 20% FBS was added to the lower chamber. After 22 h of incubation, cell culture inserts were fixed with cold methanol for 30 min and stained with 0.1% crystal violet for 30 min. Images were captured at 100× magnification, and the average number of stained cells was counted in at least five different fields. Cell invasion ability was determined using the transwell invasion assay, and each insert was precoated with BD Matrigel Basement Membrane Matrix (Corning). Cell suspensions containing 5×10^5^ cells and 200 μL of medium without FBS were cultured in the upper chamber. The bottom chamber contained 500 μL of the medium with 20% FBS. The cells were fixed with cold methanol for 30 min and stained with 0.1% crystal violet for 30 min after incubation for 22 h. The invasive cells were counted and imaged at 100× magnification. The higher number of the stained invasive cells corresponded to the stronger cell invasive ability.

Plate clone formation experiment

MDA-MB-231 and BT-549 cells transfected with and without miRNA inhibitors and mimics were counted with a cell counter. Cell suspensions (2 mL/well) with 30, 60, and 120 cells were cultured in the six-well plate and the plates were incubated for 2 weeks in a 37 ℃ cell incubator. The incubation was stopped when a white cell clone was visible. The cell clones were washed with PBS and fixed with cold methanol for 30 min. After staining with 0.1% crystal violet for 30 minutes, the number of cell clone group per well was counted under the microscope (the number of cells in a single clone should be more than 50) and the efficiency of colony formation was calculated by the following formula: formation efficiency = number of clones/number of cells inoculated. A high score indicated strong proliferation ability.

Western blot assay

The protein concentrations of MDA-MB-231 and BT-549 cells transfected with and without miRNA inhibitors and mimics, as well as tissue samples were determined. The total protein amount was quantified using a BCA Protein Assay Kit (Thermo Fisher Scientific; #23225), and then the proteins were separated using a 12.5% sodium dodecyl sulfate polyacrylamide gels and transferred to polyvinylidene fluoride membranes (Beyotime, Hamen, China). The membranes were blocked using 5% skim milk at room temperature for 2 h and then detected with rabbit anti-HHEX polyclonal (1:2000 dilution, Affinity, USA) and mouse anti-β-actin monoclonal antibody (1:1,000 dilution, Zhongshan Inc., Beijing, China). The membranes were then incubated with the appropriate secondary antibodies for 2 h at room temperature. The images were obtained using a ChemiDoc imaging system (BioRad, USA). β-actin, GAPDH, and histone H3 were used as protein loading controls. All WB analyses were repeated three times independently.

Immunohistochemical staining, scoring, and quantification

Paraffin-embedded samples were embedded in paraffin and sectioned. After antigen retrieval, tissue sections were blocked with endogenous peroxidase inhibitor and then incubated with normal goat serum to prevent nonspecific protein binding. The slides were incubated with the primary HHEX antibody was incubated at 4 ℃ for 14–16 h, and then reacted with biotinylated goat anti-mouse IgG. The signal was detected with the labeled streptavidin-biotin system in the presence of the chromogen 3,3-diaminobenzidine or alkaline phosphatase. The staining index including percentage of positive cells and the staining intensity of HHEX in human breast samples were evaluated. Brown-yellow staining of nucleus was considered a positive result. Staining intensity was scored as follows: 0, no staining; 1, faint yellow staining; 2, brownish-yellow staining; and 3, brown staining. The number of positive cells was visually evaluated and stratified as follows: 0, < 10% positive cells; 1, < 30% positive cells; 2, < 50% positive cells; and 3, > 70% positive cells. The sum percentage of positive cells and staining intensity was used to determine the staining index for each section.
