# Supplementary material for: Clinicopathological Significances of Cancer Stem Cell-Associated HHEX Expression in Breast Cancer
Source: Front Cell Dev Biol. 2020 Dec 23;8:605744. doi: 10.3389/fcell.2020.605744 (PMC7785851; doi:10.3389/fcell.2020.605744)
Supplement: Supplementary file 2 [file Data_Sheet_2.doc]

Figure S1. Flow diagram of the analysis procedure.

Data processing, identification of CSC-related genes, exploration of survival-related genes, and miRNAs.

Figure S2. Transcript levels of HHEX in BC and normal tissues

(A-B) mRNA levels of HHEX in BC determined using GEPIA. (C) mRNA levels of HHEX in BC determined using TCGAportal. (D) Overall survival differences of HHEX in BC patients determined using PROGgeneV2. T: breast cancer tissues, N: normal breast tissues.

Figure S3. HHEX Expression levels in 6 and 4 pregnancies mice breast tissues

(A) Expression levels of HHEX by western blot analysis in breast tissue of TA2 mice with 6 pregnancies and 4 pregnancies. (B) Histogram showing the quantitative results of the HHEX expression. 6: six pregnancies, 4: four pregnancies.

Figure S4. Repeat experiments for figure 2B and figure 7A. (A) The first repeat experiment for figure 2B. Expression levels of HHEX by western blot analysis in TA2 mice with different numbers of pregnancies and SBC. (B) The second repeat experiment for figure 2B. Expression levels of HHEX by western blot analysis in TA2 mice with different numbers of pregnancies and SBC. (C) The third repeat experiment for figure 2B. Expression levels of HHEX by western blot analysis in TA2 mice with different numbers of pregnancies and SBC. (D) Histogram shows the quantitative results of the HHEX expression in breast tissue TA2 mice with different numbers of pregnancies and SBC. (a) The first repeat experiment. (b) The second repeat experiment. (c) The third repeat experiment. (E) The first repeat experiment for figure 7A. (a).HHEX expression of MDA-MB-231 transfected with and without miRNA inhibitors, mimics. (b) HHEX expression of BT-549 transfected with and without miRNA inhibitors, mimics. (c). Histogram showed the quantitative result of the HHEX expression in MDA-MB-231.(d) Histogram showed the quantitative result of the HHEX expression in BT-549. (F) The second repeat experiment for figure 7A. (a).HHEX expression of MDA-MB-231 transfected with and without miRNA inhibitors, mimics. (b) HHEX expression of BT-549 transfected with and without miRNA inhibitors, mimics. (c). Histogram showed the quantitative result of the HHEX expression in MDA-MB-231.(d) Histogram showed the quantitative result of the HHEX expression in BT-549.
