# Supplementary material for: Clinicopathological Significances of Cancer Stem Cell-Associated HHEX Expression in Breast Cancer
Source: Front Cell Dev Biol. 2020 Dec 23;8:605744. doi: 10.3389/fcell.2020.605744 (PMC7785851; doi:10.3389/fcell.2020.605744)
Supplement: Supplementary file 3 [file Data_Sheet_3.doc]

Supplementary table 1. Sequences of miRNA inhibitors.

| Names | Sense (5ʹ-3ʹ) |
| --- | --- |
| miR-130b-3p | AUGCCCUUUCAUCAUUGCACUG |
| miR-301b | GCUUUGACAAUAUCAUUGCACUG |
| miR-30e-5p | CUUCCAGUCAAGGAUGUUUACA |

Supplementary table 2. miRNA mimics sequences.

| Names | Sense (5ʹ-3ʹ) | Anti-sense(5ʹ-3ʹ) |
| --- | --- | --- |
| miR-130b-3p | CAGUGCAAUGAUGAAAGGGCAU | GCCCUUUCAUCAUUGCACUGUU |
| miR-301b | CAGUGCAAUGAUAUUGUCAAAGC | UUUGACAAUAUCAUUGCACUGUU |
| miR-30e-5p | UGUAAACAUCCUUGACUGGAAG | UCCAGUCAAGGAUGUUUACAUU |

Supplementary table 3. The differences of HHEX expression in breast tissue of TA2 mice with different pregnancies and spontaneous breast cancer.

|  | Group | Gray value of HHEX expression | Value of statistic | *P* |
| --- | --- | --- | --- | --- |
| The first time | spontaneous breast cancer | 0.52±0.03 | *F= 26.93* | <0.0001 |
|  | 6 | 1.34±0.07 |  |  |
|  | 4 | 0.61±0.25 |  |  |
|  | 2 | 0.31±0.03 |  |  |
|  | 0 | 0.31±0.02 |  |  |
|  | n | 0.29±0.02 |  |  |
| The second time | spontaneous breast cancer | 0.27±0.01 | *F= 3678.0* | <0.0001 |
|  | 6 | 1.17±0.01 |  |  |
|  | 4 | 0.42±0.01 |  |  |
|  | 2 | 0.17±0.01 |  |  |
|  | 0 | 0.06±0.01 |  |  |
|  | n | 0.34±0.01 |  |  |
| The third time | spontaneous breast cancer | 0.27±0.01 | *F= 585.3* | <0.0001 |
|  | 6 | 1.34±0.06 |  |  |
|  | 4 | 0.11±0.01 |  |  |
|  | 2 | 0.07±0.01 |  |  |
|  | 0 | 0.14±0.01 |  |  |
|  | n | 0.17±0.01 |  |  |
| The fourth time | spontaneous breast cancer | 0.16±0.01 | *F= 3240.0* | <0.0001 |
|  | 6 | 1.76±0.01 |  |  |
|  | 4 | 0.46±0.02 |  |  |
|  | 2 | 0.01±0.01 |  |  |
|  | 0 | 0.18±0.08 |  |  |
|  | n | 0.14±0.01 |  |  |

n: breast tissues of virgin TA2 mice, 0: breast tissues of TA2 mice without pregnancy, 2: breast tissues of TA2 mice with 2 pregnancies, 4: breast tissues of TA2 mice with 4 pregnancies, 6: breast tissues of TA2 mice with 6 pregnancies.

Supplementary table 4. The differences of HHEX expression in MDA-MB-231 cells transfected with and without miRNA inhibitors and mimics.

|  | Group | Gray value of HHEX expression | Value of statistic | *P* |
| --- | --- | --- | --- | --- |
| The first time | NC | 0.72±0.03 | *F*= 221.5 | <0.0001 |
|  | I1 | 1.14±0.07 |  |  |
|  | I2 | 1.22±0.03 |  |  |
|  | I3 | 1.34±0.07 |  |  |
|  | M1 | 0.38±0.01 |  |  |
|  | M2 | 0.37±0.01 |  |  |
|  | M3 | 0.38±0.02 |  |  |
| The second time | NC | 0.72±0.01 | *F*= 2012.0 | <0.0001 |
|  | I1 | 1.38±0.01 |  |  |
|  | I2 | 1.72±0.01 |  |  |
|  | I3 | 0.93±0.01 |  |  |
|  | M1 | 0.83±0.01 |  |  |
|  | M2 | 0.70±0.01 |  |  |
|  | M3 | 0.69±0.03 |  |  |
| The third time | NC | 0.84±0.01 | *F*= 3544.0 | <0.0001 |
|  | I1 | 1.16±0.01 |  |  |
|  | I2 | 1.13±0.01 |  |  |
|  | I3 | 1.01±0.01 |  |  |
|  | M1 | 0.59±0.01 |  |  |
|  | M2 | 0.49±0.01 |  |  |
|  | M3 | 0.54±0.01 |  |  |

NC: normal control: M1: miR-130b mimics, M2: miR-30e mimics, M3: miR-301b mimics, I1: miR-130b inhibitor, I2: miR-30e inhibitors, I3: miR-301b inhibitor.

Supplementary table 5. The differences of HHEX expression in BT-549 cells transfected with and without miRNA inhibitors and mimics as well as normal control.

|  | Group | Gray value of HHEX expression | Value of statistic | *P* |
| --- | --- | --- | --- | --- |
| The first time | NC | 0.94±0.01 | *F*= 6423.0 | <0.0001 |
|  | I1 | 1.31±0.01 |  |  |
|  | I2 | 1.28±0.01 |  |  |
|  | I3 | 1.29±0.01 |  |  |
|  | M1 | 0.19±0.01 |  |  |
|  | M2 | 0.20±0.01 |  |  |
|  | M3 | 0.21±0.01 |  |  |
| The second time | NC | 0.91±0.02 | *F*= 2395.0 | <0.0001 |
|  | I1 | 1.02±0.02 |  |  |
|  | I2 | 1.11±0.01 |  |  |
|  | I3 | 1.23±0.03 |  |  |
|  | M1 | 0.13±0.01 |  |  |
|  | M2 | 0.11±0.01 |  |  |
|  | M3 | 0.06±0.01 |  |  |
| The third time | NC | 0.82±0.02 | *F*= 445.9 | <0.0001 |
|  | I1 | 1.42±0.03 |  |  |
|  | I2 | 1.05±0.09 |  |  |
|  | I3 | 1.62±0.04 |  |  |
|  | M1 | 0.68±0.01 |  |  |
|  | M2 | 0.17±0.01 |  |  |
|  | M3 | 0.05±0.01 |  |  |

NC: normal control cells, M1: miR-130b mimics, M2: miR-30e mimics, M3: miR-301b mimics, I1: miR-130b inhibitor, I2: miR-30e inhibitors, I3: miR-301b inhibitor.

Supplementary table 6. Comparison of migration ability in MDA-MB-231 and BT-549 cells transfected with and without miRNA inhibitors and mimics as well as normal control.

|  |  | Average migrated cells | *P* value |
| --- | --- | --- | --- |
| MDA-MB-231 | miRNA-inhibitors | 87.6667±13.0725 | 0.006 |
|  | NC | 257.0000±26.1916 |  |
|  | miRNA-mimics | 494.3333±20.8859 | 0.002 |
|  | NC | 257.0000±26.1916 |  |
| BT-549 | miRNA-inhibitors | 279.0000±23.1567 | 0.0022 |
|  | NC | 386.3333±13.7194 |  |
|  | miRNA-mimics | 625.3333±38.5602 | 0.0005 |
|  | NC | 386.3333±13.7194 |  |

Supplementary table 7. Comparison of invasion ability in MDA-MB-231 and BT-549 cells transfected with and without miRNA inhibitors and mimics as well as normal control.

|  |  | Average invasive cells | *P* value |
| --- | --- | --- | --- |
| MDA-MB-231 | miRNA-inhibitors | 99.6667±11.1156 | <0.001 |
|  | NC | 229.3333±15.1511 |  |
|  | miRNA-mimics | 401.3333±35.1125 | 0.0003 |
|  | NC | 229.3333±15.1511 |  |
| BT-549 | miRNA-inhibitors | 31.6667±6.1283 | 0.0005 |
|  | NC | 307.3333±20.9815 |  |
|  | miRNA-mimics | 556.6667±32.1904 | 0.0022 |
|  | NC | 307.3333±20.9815 |  |

Supplementary table 8. Comparison of proliferation ability in MDA-MB-231 and BT-549 cells transfected with and without miRNA inhibitors and mimics as well as normal control.

|  |  | Number of colony (%) | *P* value |
| --- | --- | --- | --- |
| MDA-MB-231 | miRNA-inhibitors | 14.6667±5.3125 | 0.0006 |
|  | NC | 56.0000±5.0990 |  |
|  | miRNA-mimics | 94.0000±5.0910 | 0.001 |
|  | NC | 56.0000±5.0990 |  |
| BT-549 | miRNA-inhibitors | 19.3333±2.6247 | 0.0007 |
|  | NC | 54.6667±5.3125 |  |
|  | miRNA-mimics | 93.0000±4.5460 | 0.0016 |
|  | NC | 54.6667±5.3125 |  |
