# Supplementary figures and images for: Clinicopathological Significances of Cancer Stem Cell-Associated HHEX Expression in Breast Cancer
Source: Front Cell Dev Biol. 2020 Dec 23;8:605744. doi: 10.3389/fcell.2020.605744 (PMC7785851; doi:10.3389/fcell.2020.605744)

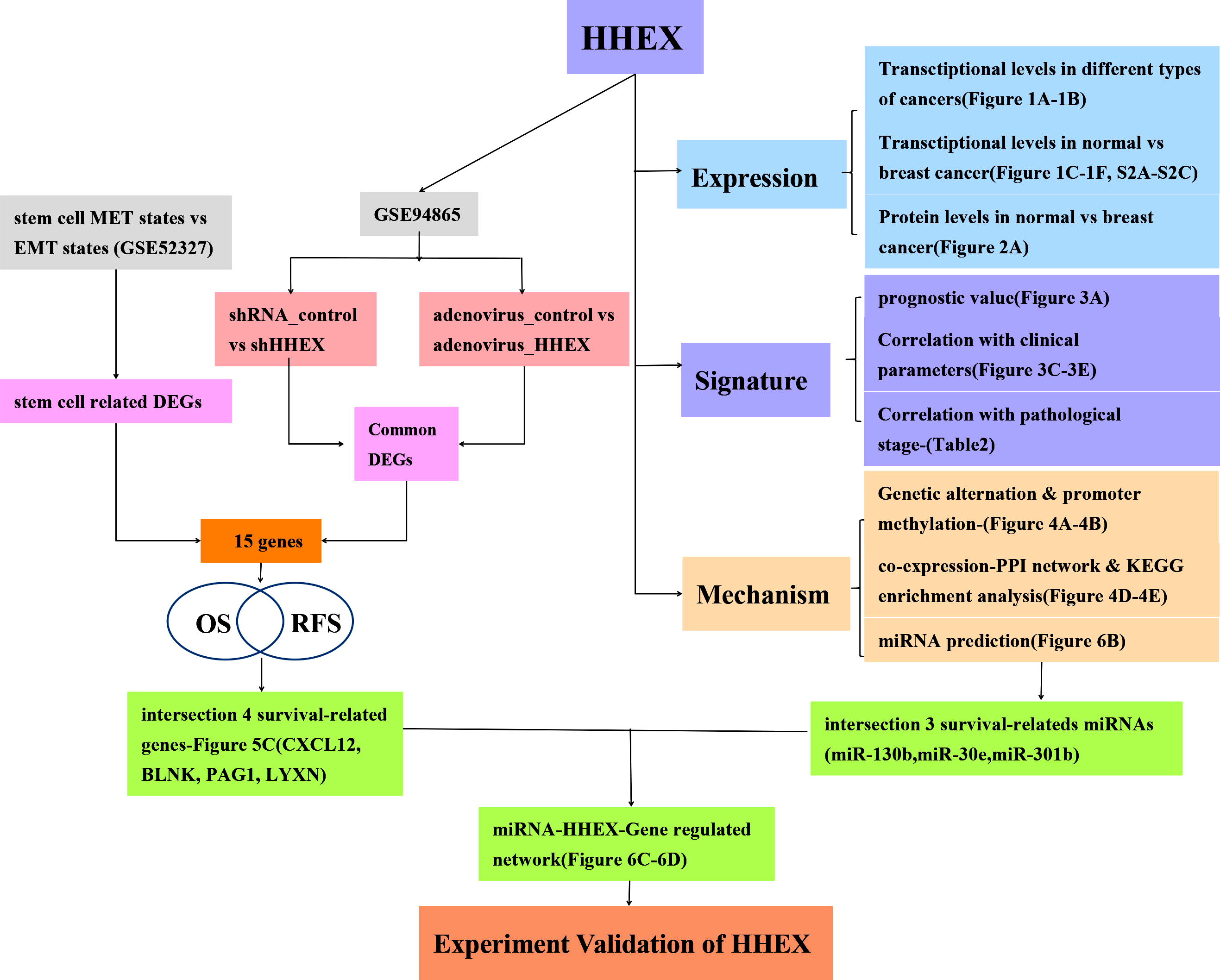

Supplement: Supplementary file 4 [file Image_1.TIF]

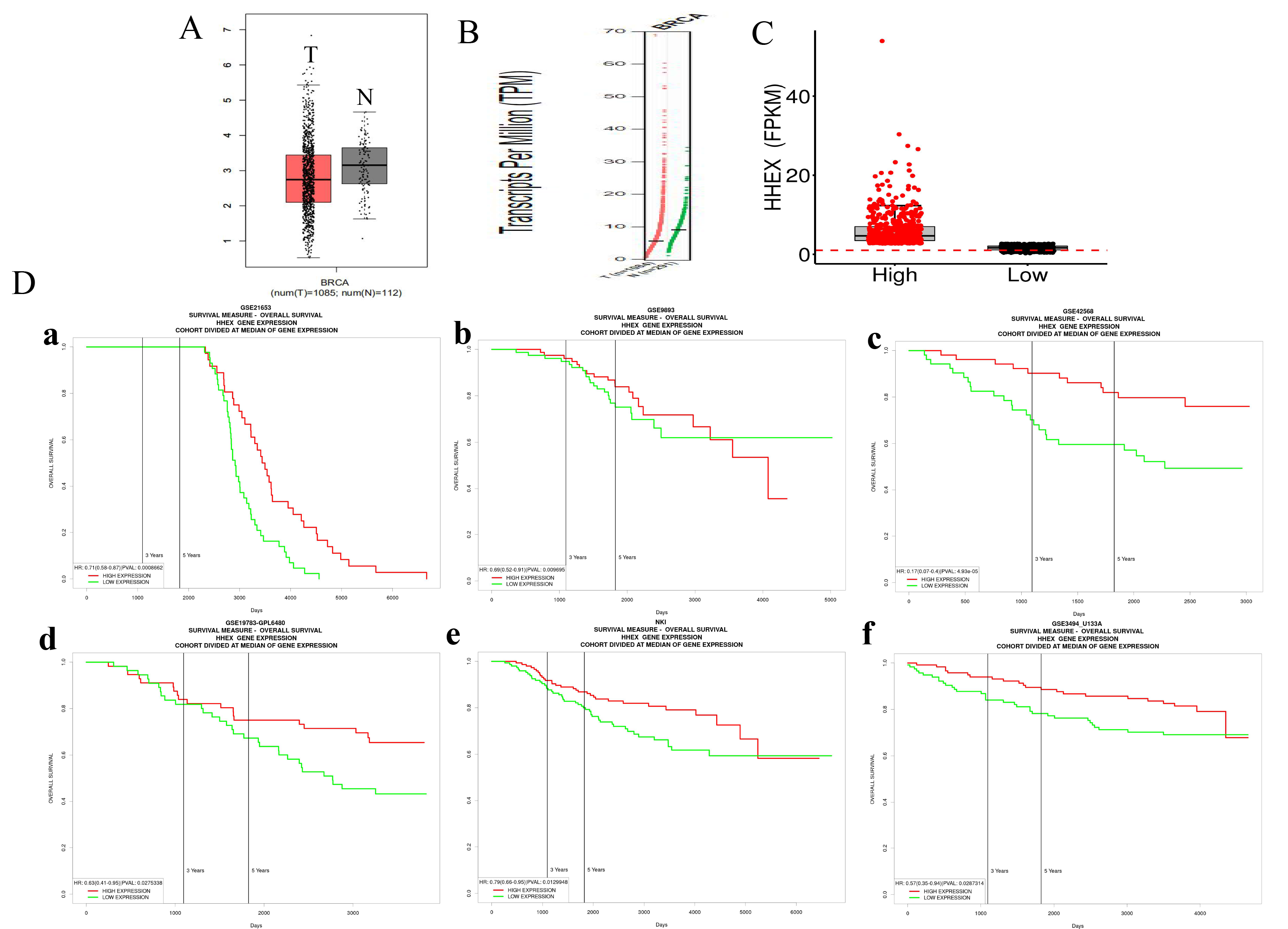

Supplement: Supplementary file 5 [file Image_2.TIF]

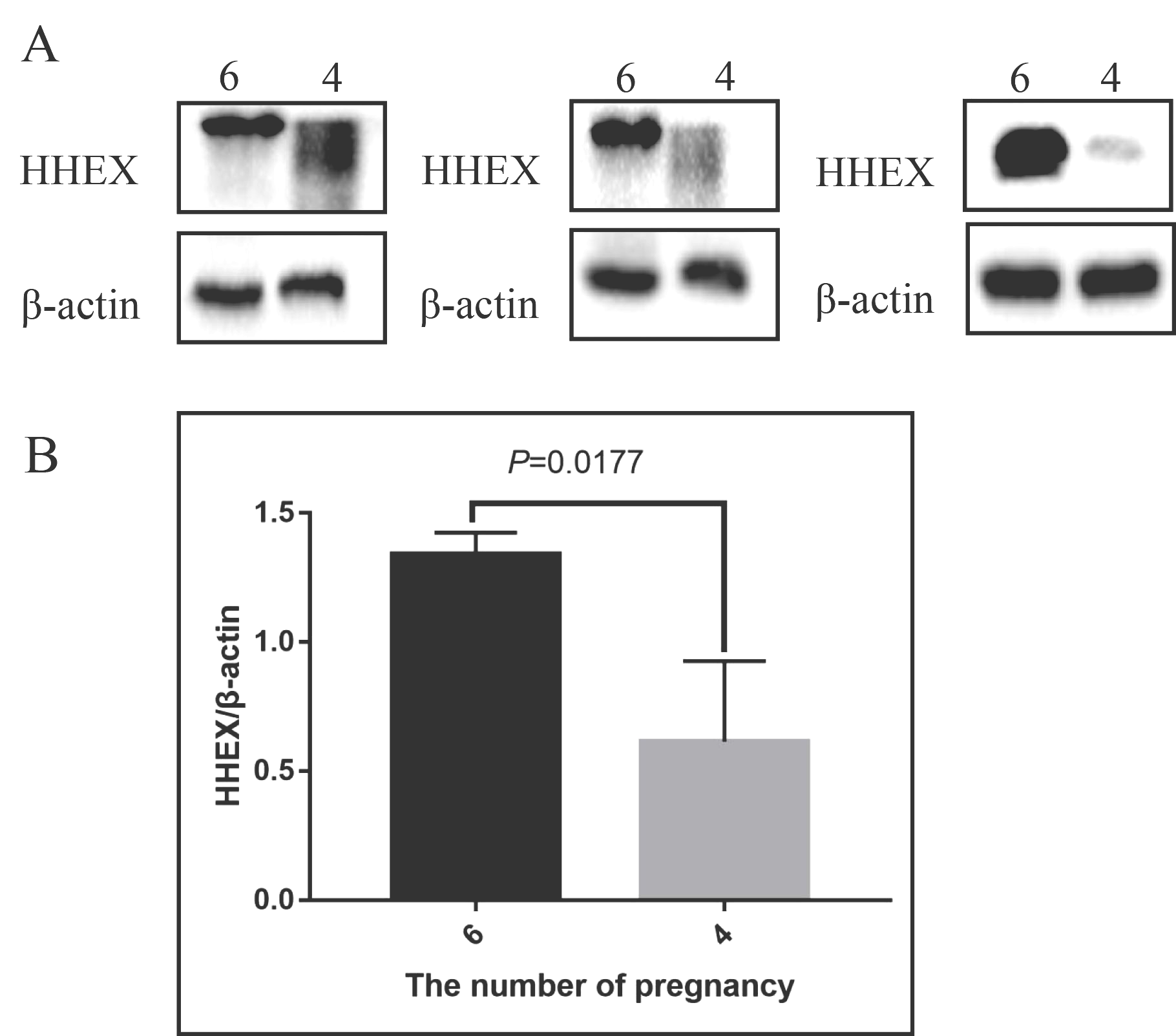

Supplement: Supplementary file 6 [file Image_3.TIF]

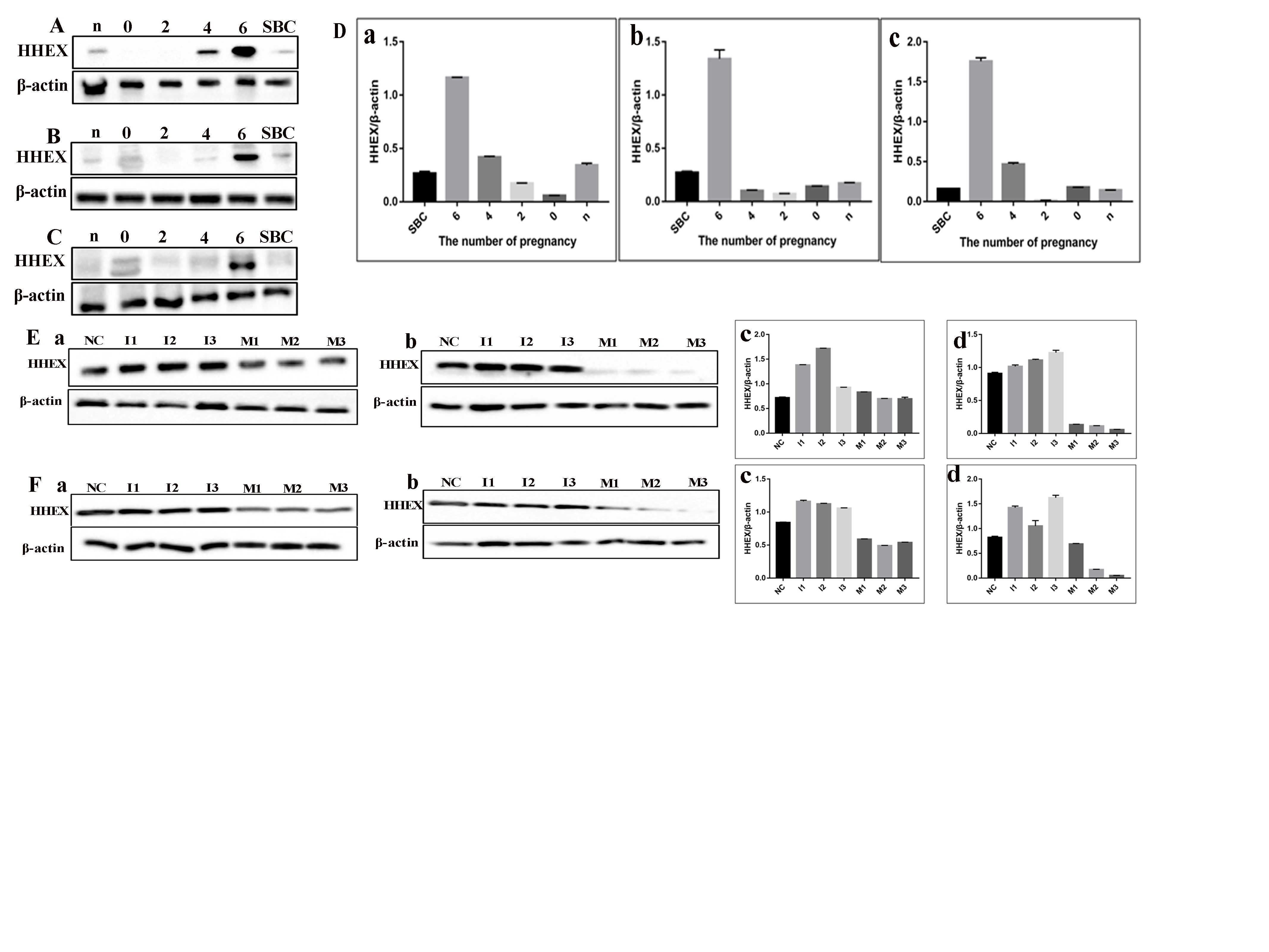

Supplement: Supplementary file 7 [file Image_4.TIF]
